# Reporter Molecules Embedded Au@Ag Core-Shell Nanospheres as SERS Nanotags for Cardiac Troponin I Detection

**DOI:** 10.3390/bios12121108

**Published:** 2022-12-01

**Authors:** Ding Wang, Yiru Zhao, Shen Zhang, Liping Bao, Huijun Li, Jingcheng Xu, Bin He, Xumin Hou

**Affiliations:** 1 School of Materials and Chemistry, University of Shanghai for Science and Technology, Shanghai 200093, China; 2 Department of Critical Care Medicine, Shanghai Chest Hospital, Shanghai Jiao Tong University School of Medicine, Shanghai 200092, China

**Keywords:** Au@Ag nanospheres, GERTs, SERS, cTn I detection, AMI

## Abstract

Rapid and accurate detection of acute myocardial infarction can improve patients’ chances of survival. Cardiac troponin I (cTn I) is an important diagnostic biomarker for acute myocardial infarction. However, current immunoassays are insufficient to accurately measure cTn I, as they have limited detection sensitivity and are time-consuming. Surface-enhanced Raman scattering (SERS) is a brilliant fingerprints diagnostic technique characterised by ultrasensitivity, fast response, and qualitative and quantitative analysis capabilities. In this study, reporter molecules (4-Mercaptobenzoic acid, 4-MBA) embedded Au@Ag core-shell nanospheres as SERS nanotags were prepared for the detection of cTn I. As the Raman reporters were embedded between the core and the shell, they could be protected from the external environment and nanoparticle aggregation. Excellent SERS performances were obtained due to the enhanced local electromagnetic field in the gap of core and shell metals. In a standard phosphate buffered saline (PBS) environment, the limit of detection for cTn I was 0.0086 ng mL^−1^ (8.6 ppt) with a good linear relationship. The excellent Raman detection performance was attributed to the localized surface plasmon resonance effect and strong electromagnetic field enhancement effect produced by the gap between the Au core and the Ag shell. The SERS nanotags we prepared were facile to synthesize, and the analysis procedure could be completed quickly (15 min), which made the detection of cTn I faster. Therefore, the proposed SERS nanotags have significant potential to be a faster and more accurate tool for acute myocardial infarction diagnostics.

## 1. Introduction

Acute myocardial infarction (AMI) is one of the most common types of cardiovascular disease. Blockage of a coronary artery, lack of blood supply (ischemia), and myocardial muscle being damaged can all lead to an AMI [1]. As myocardial necrosis is irreversible, accurate diagnosis and timely treatment are essential to improve survival rates. Cardiac troponin I (cTn I)’s outstanding specificity and supreme sensitivity to acute myocardial cell damage are why it is preferred for diagnosis; this biomarker is considered to be the gold standard of AMI diagnosis [2,3].

At present, many immunoassay methods, including enzyme-linked immunosorbent assay (ELISA) [4], electrochemistry [5], fluorescence, radioimmunoassay (RIA) [6], colloidal gold immunochromatography [7], electrochemiluminescence [8], and surface-enhanced Raman spectroscopy (SERS) [9,10] are employed for the detection of cTn I in patient serum. Although quantitative detection could use the immunochemical methods mentioned above, the disadvantages are that the specific operational steps are too cumbersome, the detection time is too long, and some experimental operations have radioactive pollution. In addition, both chemiluminescence and spectrophotometry have high requirements for processing and equipment, as well as other test conditions. Notably, SERS has received significant attention in biology and medicine because of its ultrasensitivity, fast response, and qualitative and quantitative analysis [11,12]. Zhang et al. proposed a lateral flow assay (LFA) based on core-shell surface-enhanced Raman scattering (SERS) nanotags for multiplex and quantitative detection of cardiac biomarkers for the early diagnosis of acute myocardial infarction (AMI) [13]. As they are a key aspect of SERS technology, research regarding substrate materials has attracted more and more attention.

Precious metal nanoparticles can produce a strong electromagnetic field enhancement effect, resulting in localized surface plasmon resonance (LSPR) under Raman laser. Noble metals were the most widely studied SERS substance. In the early stages, noble metal (Au, Ag and Cu) nanomaterials were developed primarily around a single noble metal [14], from nanoparticle balls and nanorods. Later, noble metals regulated other morphologies, such as nanostars [15], nanocones [16], nanocubes [17], and nanotriangles [18]. However, the performance of a single noble metal is greatly limited in practical applications. Moreover, the application of a single noble metal system in SERS biology was greatly limited due to its poor stability and how easily it aggregates.

Gap-enhanced Raman tags (GERTs) are regarded as an emerging class of SERS tags. GERTs can generate 1–2 orders of magnitude SERS response within the nanoscale gap between the plasmonic core and shell [19,20,21]. Additionally, Raman reporters are prevented from being lost because they are enclosed between the core-shell structures. Therefore, adding a protective layer between noble metals and biomolecules could avert the loss of Raman reporters and prevent the contamination of potential material signals in the surrounding medium. The usual protective layers include biomolecules, liposomes, and polymers. Among these, liposomes have high inherent biocompatibility, good stability, excellent self-assembly, and targeting ability. Bovine serum albumin (BSA) is the most widely used biomolecule protective layer, with a molecular weight of approximately 66.430 kDa. It can be effectively adsorbed on the metal surface, which not only improves the stability of metal nanomaterials and Raman reporters, but also prevents the loss of biological characteristics caused by direct contact between target antigens and metal nanomaterials [22,23]. Ma et al. reported a new strategy to form Au superparticles with high SERS enhancement via one-pot formation and self-assembly of Au nanoparticles. The self-assembly of the Raman reporter on Au superparticles generated SERS nanotags with intense signals [24]. Liposomes were also applied in a biodegradable photothermal and pH-responsive calcium carbonate@phospholipid@acetylated dextran hybrid platform to advance biomedical applications [25]. However, the steps in the experimental process were exceeding cumbersome, which limited its application. Therefore, novel GERTs for more sensitive and faster bioinstrumentation need to be investigated.

In this study, gap-enhanced Raman tags with 4-Mercaptobenzoic acid (4-MBA) embedded Au@Ag core-shell nanospheres (NSs) were designed. Raman reporters were embedded between the core-shell interspace and could be protected from the external environment and nanoparticle aggregation. The structure, morphology, and composition of Au@Ag NSs were characterized and studied. The SERS performance of the nanotags for detecting cTn I was evaluated. Furthermore, the bovine serum albumin used as a protective layer was modified on the surface of nanospheres to prevent the contamination of potential material signals in the surrounding medium. Finally, the enhancement of an electromagnetic field was simulated using COMSOL. The results showed that the remarkable SERS performance of nanotags was related to the electromagnetic field enhancement mechanism. It may provide a novel exploration for acute myocardial infarction diagnosis.

## 2. Materials and Methods

### 2.1. Materials and Reagents

All materials and reagents were of analytical grade and used without further purification. The water was ultrapure deionized water. The materials and reagents used in this study were chloroauric acid (HAuCl_4_, ≥96%); ascorbic acid (C_6_H_8_O_6_, ≥99.7%); silver nitrate (AgNO_3_, ≥99.8%) was purchased from Sinopcc Chemical Reagent Co., Ltd. (Shanghai, China); ethanol (≥99.7%); hexadecyl trimethyl ammonium chloride (C_19_H_42_ClN, ≥99%) (Adamas); 4-Mercaptobenzoic acid (4-MBA, ≥95%) was purchased from Tixiai Chemical Industrial Development Co., Ltd. (Shanghai, China); sodium citrate (C_6_H_5_Na_3_O_7_, ≥99%); EDC and NHS were purchased from Shanghai Aladdin Biochemical Technology Co., Ltd. (Shanghai, China); BSA (MW = 66 kDa) was purchased from Yeasen Biotech Co., Ltd. (Shanghai, China); COOH-PEG-SH (Mw = 5000) was purchased from Yuan Ye Biological Co., Ltd. (Shanghai, China); phosphate buffered saline (PBS) 1× concentrate (Adamas); anti-cTn I (L3C00405); and recombinant human cTn I antigen (L4C00102) (Linc-Bio).

### 2.2. Preparation of Au@Ag Nanospheres

Preparation of Au NSs: Au NSs were synthesized using a modified liquid reduction method [26]. First, 50 mL of 0.01 wt. % HAuCl_4_ solution was placed into a round-bottom flask and heated in a water bath until boiling, followed by rapid addition of 1.0 mL of 1 wt. % sodium citrate solution under magnetic stirring. After 30 min of reaction, the Au NSs were prepared.

Preparation of Au@Ag NSs: First, 2 mL of Au seed solution and 40 mL of deionized water were added into a round-bottom flask under magnetic stirring. Second, 2 mL of 1 wt. % sodium citrate solution and 2 mL of 20 mmol·L^−1^ ascorbic acid solution were added into the mixture and stirred for 5 min. Third, 0.5 mL of 10 mmol·L^−1^ silver nitrate (AgNO_3_) solution was added dropwise into the mixture and stirred for 15 min. During this procedure, the color of the mixture changed from wine red to orange to golden yellow.

### 2.3. Synthesis of Au@4-MBA@Ag SERS Nanotags

First, 2 mL of Au seed solution was added to 10 mL of 0.1 M cetyltrimethylammonium chloride (CTAC) solution and sonicated for 5 min. Second, 50 μL of 60 mM 4-MBA solution was added into the mixture and sonicated for 30 min. Third, 1 mL of 40 mM ascorbic acid (AA) and 1 mL of 10 mM AgNO_3_ aqueous solution were added successively. After reacting for 60 min, the mixture was washed via centrifugation with water. The Au@4-MBA@Ag was redispersed into the water for use.

Next, 2 mL of the above solution was added to 100 μL of 500 μM SH-PEG-COOH, and reacted at 4 °C overnight. At room temperature (25 °C), 10 μL of 40 mg/mL EDC and 10 μL of 110 mg/mL NHS were added and shaken in the dark for 15 min. Next, 10 μL of the antibody at a concentration of 0.1 ng/mL was added, and the reaction was incubated in an oven at 37 °C for 1 h. Lastly, 10 μL of BSA at a concentration of 1 mg/mL was incubated in an oven at 3 °C for 30 min. The SERS nanotags were stored at 4 °C for use.

### 2.4. Materials Characterization and SERS Test

Scanning electron microscopy (SEM) was performed using an FEI Quanta 400 instrument. HRTEM measurements were carried out using a JEOL 2100 HRTEM. The topography and thickness of samples were characterized using an atomic force microscope (AFM). A Lambda 750S ultraviolet visible spectrometer was used for ultraviolet visible spectrum analysis. Raman spectra measurements were carried out using a Raman Spectrograph; 10 μL of standard PBS antigen cTn I-Antigen and 10 μL of cTn I-Antibody-labeled SERS nanotags were instilled in the grooves of the slide’s glass and incubated at 37 °C for 15 min. The parameters of the SERS test were laser wavelength, 633 nm; spectra, in the range of 500–2000 cm^−1^; and integral time, 10 s.

## 3. Results and Discussion

**Figure 1:**
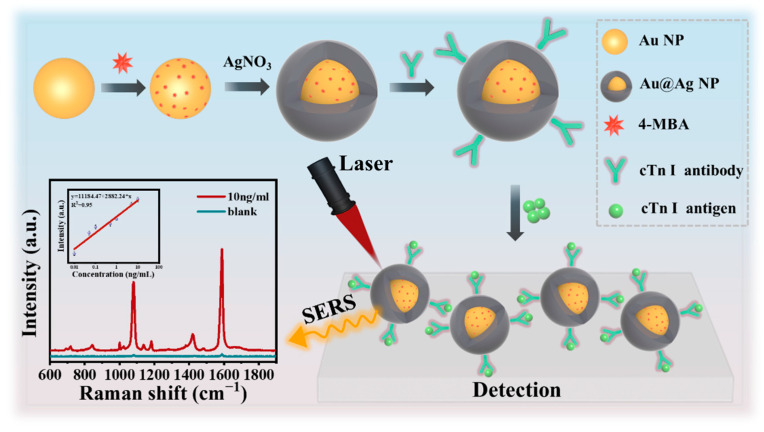
Scheme for the preparation of SERS nanotags and the detection of cTn I.

To form a mass of “hot spot”, SERS nanotags were prepared for quantitative and ultrasensitive detection of cTn I. As shown in Figure 1, 4-MBA was embedded in the gap between Au and Ag bimetallic nanospheres to form Au@4-MBA@Ag, which was further combined with antibody to form an immune probe used for cTn I detection. The preparation and detection of the SERS biosensor can be described as follows. First, noble metal gold nanospheres (Au NSs) were synthesized using a modified liquid reduction method, and Raman reporters were modified on the surface of the Au core [27]. Second, the core-shell composite material Au@Ag NSs combined with 4-MBA was prepared by growing Ag shells on the surface of Au@4-MBA. To form Ag-S chemical bonds, carboxyl polyglycol sulfhydryl group was modified using Ag shells, then NHS and EDC solution were injected to activate carboxyl to connect with cTn I antibody. Third, the cTn I antibody was combined with the mixture to prepare specific SERS nanotags. Finally, SERS nanotags were used to capture antigens. Compared with Hu et al., in the absence of magnetic beads, we only used simple synthetic SERS nanotags to complete the immune test, and the detection time was reduced to 15 min [27]. In the detection process, under laser irradiation, the Raman spectrum of the SERS reporters changed according to changes in antigen concentration; then linear fitting was carried out according to changes in its relative peak intensity and antigen concentration to obtain a curve with a good linear relationship.

### 3.1. Morphological and Raman Characterization of Au and Au@Ag

**Figure 2:**
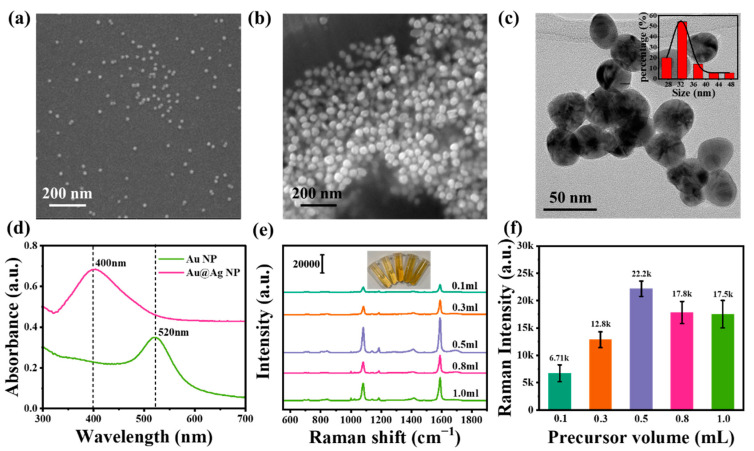
(**a**) SEM image of Au NSs; (**b**) SEM image of Au@Ag NSs in 0.5 mL AgNO_3_; (**c**) HRTEM image of Au@Ag NSs in 0.5 mL AgNO_3_, with inset showing data from the statistical analysis of Au@Ag NSs size (fitted with a Gaussian distribution); (**d**) UV-vis spectra of Au NSs and Au@Ag NSs; (**e**) Raman spectra of 60 mM 4-MBA on Au@Ag with different volumes of AgNO_3_ (from 0.1 mL to 1.0 mL); (**f**) histogram of the standard error statistics of Raman intensity as the volume of AgNO_3_ was increased from 0.1 mL to 1.0 mL.

The morphologies of Au and Au@Ag were characterized using scanning electron microscopy (SEM) and transmission electron microscopy (TEM). As shown in Figure 2a, the Au NSs had uniform size and good dispersion, and the nanosphere size was approximately 15 nm. However, the formation of Ag shells on the surface of Au NSs had no effect on the size of pristine Au NSs. As shown in Figure 2b, Au@Ag NSs with uniform morphology and dispersion were successfully prepared; the obvious core-shell structure can be seen in the TEM image of Figure 2c. Au NSs and Au@Ag NSs were successfully synthesized. In Figure 2d, Au NSs have an obvious absorption peak at approximately 520 nm, which corresponded to the plasmon resonance absorption peak of Au [28,29], and Au NSs exhibited a dipolar LSPR mode at 520 nm. After the Ag shell was grown on the surface of Au NSs, the peak position of the absorption peak shifted to 400 nm, corresponding to the plasmon resonance absorption peak of Ag [30]. Obviously, the Au peak almost disappeared and the Ag peak dominated; this was caused by the high content of Ag in the core-shell structure. Furthermore, Ag NSs exhibited a dipolar LSPR mode at 400 nm, and thus confirmed the formation of Ag NSs. To compare the enhancement effect of different Ag contents on Raman signals, Au@Ag Raman spectra prepared using different AgNO_3_ contents were compared under the same measurement conditions. As shown in Figure S2, we compared the Raman spectra of pure Au NPs, 4-MBA and pure Au NPs + 4-MBA. As shown in Figure 2e, the Raman intensity gradually increased with the AgNO_3_ from 0.1 mL to 0.5 mL, but decreased by approximately 4400 when the volume of AgNO_3_ reached 0.8 mL. After the volume of AgNO_3_ was further increased to 1.0 mL, the intensity decreased by approximately 4700. Furthermore, as shown in Figure 2f, when the volume of AgNO_3_ was increased from 0.5 mL to 1.0 mL, the standard error still maintained an upward trend, indicating a further decrease in stability and reliability. Therefore, 0.5 mL was chosen as the optimal volume of AgNO_3_ for synthesis, other characterization methods, and performance testing.

### 3.2. Characterization of Core-Shell Structure

**Figure 3:**
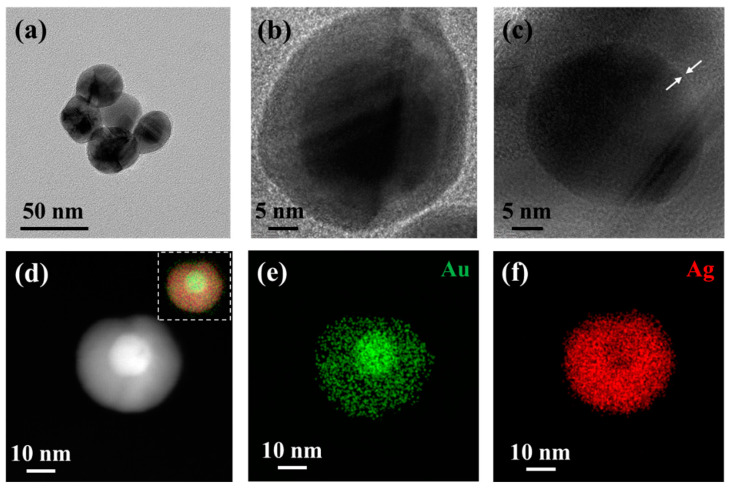
(**a**,**b**) HRTEM images of Au@Ag NSs; (**c**) HRTEM image of Au@4-MBA@Ag NSs; (**d**,**e**) images and EDS elemental maps of Au@Ag (inset image is superimposed image of Au and Ag signals).

The morphology of core-shell materials was observed more carefully using transmission electron microscopy (TEM). As shown in Figure 3a,b, after adding AgNO_3_ solution to deposit the silver shell, double-layer nanospheres with a size of 30 nm could be clearly observed by high-resolution TEM imaging (Figure 3b). The boundary was obvious because the electron density of the darker part, corresponding to Au NSs, and the lighter part, corresponding to the silver deposited around Au NSs, were different. In the image in Figure 3a, the particles showed a uniformly grown Au@Ag core-shell structure. The Raman reporters were immobilized by adding the Raman signal molecule 4-MBA in the interspace of the Au@Ag NSs core-shell structure for later quantitative analysis. As shown in Figure 3c, a small gap in the middle of the core-shell structure was attributed to the molecular layer formed by 4-MBA. In addition, the EDS element maps in Figure 3e,f show the core-shell structure formed by Au and Ag; it was nucleated with Au element, and Ag element grew uniformly on the surface of Au.

### 3.3. SERS Performance

**Figure 4:**
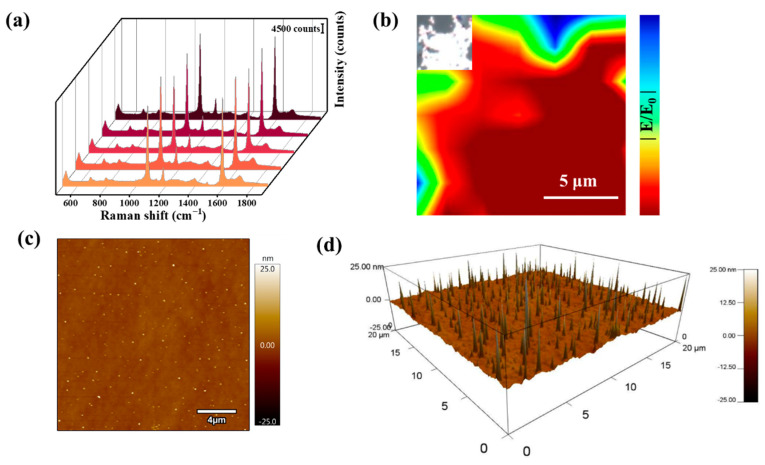
(**a**) 3D Raman spectra of Au@Ag; (**b**) 2D Raman imaging of Au@Ag (inset is the optical image of Au@Ag); AFM images of (**c**) 2D mode and (**d**) 3D mode of Au@Ag.

The SERS performance of Au@4-MBA@Ag NSs was investigated. The uniformity of Au@Ag was studied using a Laser Raman Microscope, as shown in the inset of Figure 4b. As shown in Figure S3, we studied the three-dimensional Raman spectra of Au. The three-dimensional waterfall spectra (Figure 4a) of the Au@Ag from Raman mapping and the uniformity of the SERS relative intensity were obtained as two-dimensional maps (based on I_1078_). Figure 4b shows that Au@Ag as a SERS substrate possessed excellent signal spatial stability and consistency, which was in good agreement with the uniform morphology of the SERS substrate (Figure 2b). The overall morphology of the SERS substrate was further observed using atomic force microscopy (AFM). This was also consistent with the performance test results shown in Figure 4a and the SEM results in Figure 2b, which finally showed that the overall morphology of the SERS substrate was uniform and that the SERS performance was stable.

**Figure 5:**
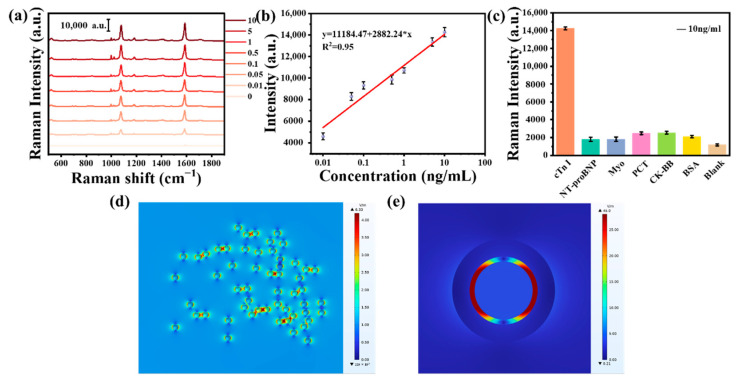
(**a**) Raman spectra of cTn I antigen samples with different concentrations on SERS nanotags; (**b**) linear relationship between Raman signal and cTn I antigen concentration; (**c**) Raman signals of different biomarkers to the SERS nanotags; (**d**) electrical field distributions of randomly distributed Au NSs; and (**e**) Au@Ag (633 nm laser wavelength).

The capability of the SERS nanotags for detecting biomarker cTn I was further investigated. A series of cTn I standard solutions with different concentrations (0, 0.01, 0.05, 0.1, 0.5, 1.0, 5.0, and 10.0 ng mL^−1^) were prepared and tested under the same conditions. As shown in Table S1, we compared the detection limits of cTn I by different detection methods. The Raman spectra of the standard PBS troponin antigen solution is show in Figure 5a. The Raman signal was gradually enhanced via an increase in cTn I antigen concentration. As shown in Figure 5b, a relative curve was fitted to the Raman signal and cTn I antigen concentration. Correspondingly, the equation for the fitting curve of cTn I was I = 11184.47 + 2882.24 ∗ C (R^2^ = 0.95), and the range was 0.01–10.0 ng mL^−1^ (Figure 5b). The specificity of SERS nanotags to cTn I was also tested. Under the same test conditions, the Raman response of nanotags to cTn I was significantly higher than the responses of other myocardial infarction biomarkers and blank experiments, indicating that the Au@Ag Biosensors had excellent specificity.

The gap between the gold core and silver shell can produce a strong electromagnetic field enhancement effect, resulting in plasmon resonance under a 633 nm laser [31]. The simulation was conducted using COMSOL under a 633 nm laser. As shown in Figure 5d, the electromagnetic field enhancement of randomly distributed Au NSs could reach a maximum of 6.33, whereas the electromagnetic field enhancement of Au@Ag NSs with GERTs could reach a maximum of 45.0. As shown in Figure S1, we compare the electromagnetic field enhancement effect under a 532 nm laser. The COMSOL simulation results provided strong support for the interpretation of the Au@Ag electromagnetic field enhancement mechanism.

## 4. Conclusions

In summary, the Au@4-MBA@Ag NSs core-shell structure was successfully prepared. The Au@4-MBA@Ag NSs core-shell structure with excellent signal consistency and steric stability was considered as a SERS substrate. In addition, the biosensor based on Au@Ag could be used for cTn I detection in a standard phosphate buffered saline (PBS) environment; the limit of detection for cTn I was 0.0086 ng mL^−1^ with a good linear relationship. The COMSOL simulation results indicated the electromagnetic field enhancement mechanism of Au@Ag core-shell structure and the enhancement of the Raman reporter. Additionally, the Au@4-MBA@Ag NSs core-shell structure was facile to synthesize, and the analysis procedure could be completed within 15 min. Therefore, the biosensor based on Au@Ag has great potential to be a fast and accurate detection tool for cTn I and an effective diagnostic tool, as well as significant application prospects in clinical applications.

## Data Availability

Not applicable.

## Supplementary Materials

The following supporting information can be downloaded at: https://www.mdpi.com/article/10.3390/bios12121108/s1, Figure S1. (a) Electrical field distributions of randomly distributed Au NSs and (b) Au@Ag NS with GERTs (532 nm laser wavelength); Figure S2. Raman spectra of pure Au NPs, 4-MBA and pure Au NPs + 4-MBA; Figure S3. Three-dimensional Raman spectra of Au; Table S1. Detection limits of cTn I by different detection methods [32,33,34,35,36,37].
