# Supplementary material for: Reporter Molecules Embedded Au@Ag Core-Shell Nanospheres as SERS Nanotags for Cardiac Troponin I Detection
Source: Biosensors (Basel). 2022 Dec 1;12(12):1108. doi: 10.3390/bios12121108 (PMC9775458; doi:10.3390/bios12121108)
Supplement: Supplementary file 1 [file biosensors-12-01108-s001.zip › biosensors-1995688-supplementary.pdf]

biosensors

Supplementary Files

Reporter molecules embedded Au@Ag core-shell nanospheres as SERS nanotags for cardiac troponin I detection

Ding Wang[1,2], Yiru Zhao[1], Shen Zhang[2], Liping Bao[1], Huijun Li[1], Jingcheng Xu[1], Bin He [2,*] and Xumin Hou[2,*]

1 School of Materials and Chemistry, University of Shanghai for Science and Technology, 516 Jungong Road, Shanghai 200093, China.
2 Department of Critical Care Medicine, Shanghai Chest Hospital, Shanghai Jiao Tong University School of Medicine, Shanghai, 200092, China.
* Correspondence: bin_he@sjtu.edu.cn (B. H.); houxumin@sjtu.edu.cn (XM. H.);

The calculation procedure of electromagnetic field distribution: Firstly, the geometric models were established according to the morphology of Au NSs and Au@Ag NS. Secondly, the material properties of gold and silver were assigned. Then, the frequency domain of the electromagnetic field was set and the scattering field was used in the calculation, and the parameter of scattering field is E0*exp(-j*ewfd.k0*z). Finally, the frequency response of the corresponding wavelength is calculated. (Other parameters: Pressure (Pa): one atmosphere. Temperature (K): 293.15 K. Laser wavelength: 532 nm)

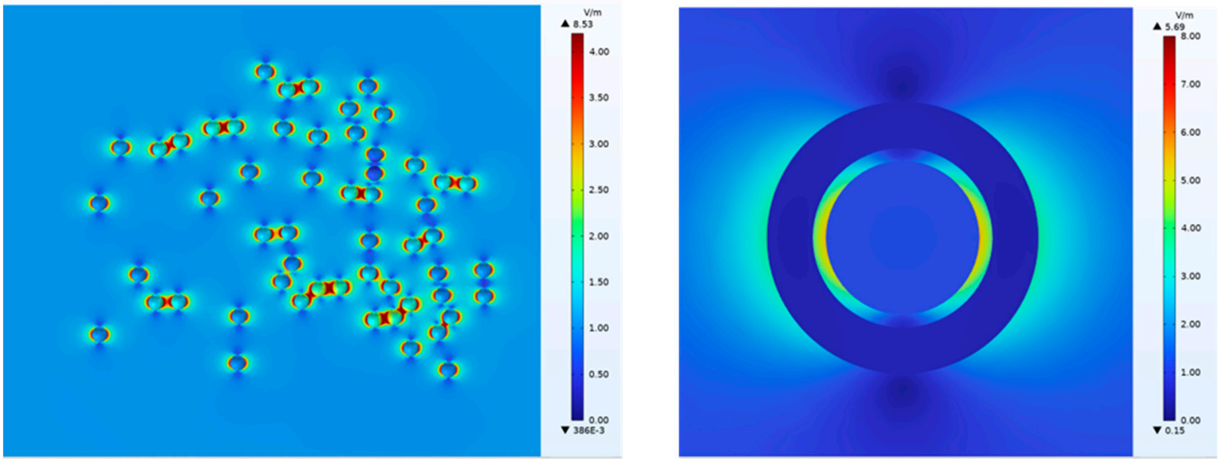

Figure S1. (a) Electrical field distributions of randomly distributed Au NSs and (b) Au@Ag NS with GERTs.

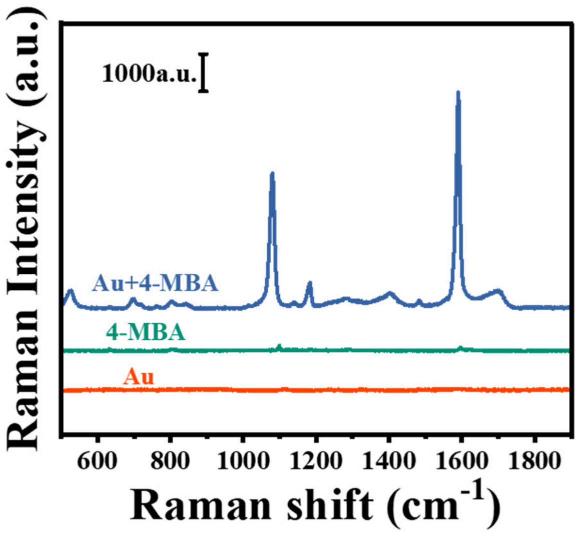

Figure S2. Raman spectra of pure Au NPs 、4-MBA and pure Au NPs+4-MBA.

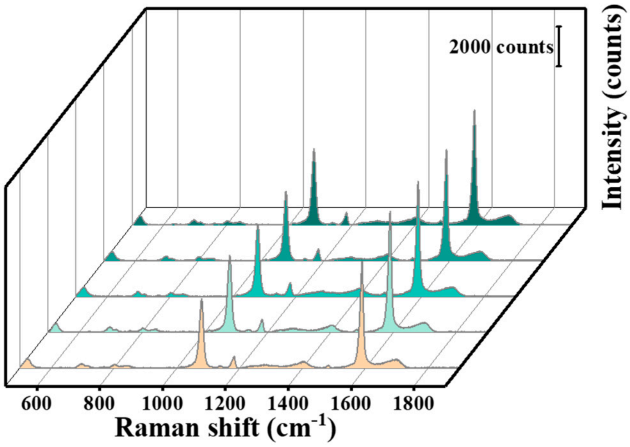

Figure S3. Three-dimensional Raman spectra of Au.

Table S1. Detection limits of cTn I by different detection methods.

Methods	Linear ranges （ng/mL）	LOD （pg/mL）	Reference
Electrochemical Immunoassay	0.5 ~ 100	40	[1]
ELISA	0.1 ~ 10	27	[2]
fluorescence	3.9 ~ 100	980	[3]
Photoelectrochemical	50 ~ 500000	8.0	[4]
Differential Pulse Voltammetry	1.25 ~ 125	67.5	[5]
Enzymatic Chemiluminescence	0.1 ~ 50	50	[6]
SERS	0.01 ~ 10	8.6	This work

1. Rezaei, B.; Shoushtari, A.M.; Rabiee, M.; Uzun, L.; Mak, W.C.; Turner, A.P.F. An electrochemical immunosensor for cardiac Troponin I using electrospun carboxylated multi-walled carbon nanotube-whiskered nanofibres. *Talanta*. **2018**, 182, 178-186.
2. Cho, I.-H.; Paek, E.-H.; Kim, Y.-K.; Kim, J.-H.; Paek, S.-H. Chemiluminometric enzyme-linked immunosorbent assays (ELISA)-on-a-chip biosensor based on cross-flow chromatography. *Analytica Chimica Acta.* **2009**, 632, 247-255.
3. Toma, K.; Oishi, K.; Iitani, K.; Arakawa, T.; Mitsubayashi, K. Surface plasmon-enhanced fluorescence immunosensor for monitoring cardiac troponin I. *Sensors and Actuators B-Chemical.* **2022**, 368.
4. Negahdary, M.; Behjati-Ardakani, M.; Sattarahmady, N.; Yadegari, H.; Heli, H. Electrochemical aptasensing of human cardiac troponin I based on an array of gold nanodumbbells-Applied to early detection of myocardial infarction. S*ensors and Actuators B-Chemical.* **2017**, 252, 62-71.
5. Zuo, J.; Zhao, X.; Ju, X.; Qiu, S.; Hu, W.; Fan, T.; Zhang, J. A New Molecularly Imprinted Polymer (MIP)-based Electrochemical Sensor for Monitoring Cardiac Troponin I (cTnI) in the Serum. *Electroanalysis.* **2016**, 28, 2044-2049.
6. Liu, J.; Zhang, L.; Wang, Y.; Zheng, Y.; Sun, S. An improved portable biosensing system based on enzymatic chemiluminescence and magnetic immunoassay for biological compound detection. *Measurement.* **2014**, 47, 200-206.
